# Glutamine supplementation reduces markers of intestinal permeability during running in the heat in a dose-dependent manner

**DOI:** 10.1007/s00421-017-3744-4

**Published:** 2017-10-20

**Authors:** Jamie N. Pugh, Stephen Sage, Mark Hutson, Dominic A. Doran, Simon C. Fleming, Jamie Highton, James P. Morton, Graeme L. Close

**Affiliations:** 1 Research Institute for Sport and Exercise Sciences, Liverpool John Moores University, Tom Reilly Building, Byrom Street, Liverpool, L3 3AF UK; 2 Royal Cornwall Hospital, Truro, UK; 3 Department of Sport and Exercise Sciences, University of Chester, Chester, UK

**Keywords:** Glutamine, Exercise, Intestinal permeability, Gastrointestinal symptoms

## Abstract

**Purpose:**

To examine the dose–response effects of acute glutamine supplementation on markers of gastrointestinal (GI) permeability, damage and, secondary, subjective symptoms of GI discomfort in response to running in the heat.

**Methods:**

Ten recreationally active males completed a total of four exercise trials; a placebo trial and three glutamine trials at 0.25, 0.5 and 0.9 g kg^−1^ of fat-free mass (FFM) consumed 2 h before exercise. Each exercise trial consisted of a 60-min treadmill run at 70% of $\dot {V}{{\text{O}}_{{\text{2max}}}}$ in an environmental chamber set at 30 °C. GI permeability was measured using ratio of lactulose to rhamnose (L:R) in serum. Plasma glutamine and intestinal fatty acid binding protein (I-FABP) concentrations were determined pre and post exercise. Subjective GI symptoms were assessed 45 min and 24 h post-exercise.

**Results:**

Relative to placebo, L:R was likely lower following 0.25 g kg^−1^ (mean difference: − 0.023; ± 0.021) and 0.5 g kg^−1^ (− 0.019; ± 0.019) and very likely following 0.9 g kg^− 1^ (− 0.034; ± 0.024). GI symptoms were typically low and there was no effect of supplementation.

**Discussion:**

Acute oral glutamine consumption attenuates GI permeability relative to placebo even at lower doses of 0.25 g kg^−1^, although larger doses may be more effective. It remains unclear if this will lead to reductions in GI symptoms. Athletes competing in the heat may, therefore, benefit from acute glutamine supplementation prior to exercise in order to maintain gastrointestinal integrity.

## Introduction

Gastrointestinal (GI) discomfort is frequently reported by endurance athletes in long-distance events such as marathons and triathlons (Gil et al. 1998). Indeed, 30 to 65% of long-distance runners experience some deleterious GI symptoms related to exercise including nausea, vomiting, abdominal cramps and the urge to have a bowel movement (Riddoch and Trinick 1988). However, the precise mechanisms underpinning such GI complaints during prolonged endurance exercise are not yet fully understood. Decreased splanchnic perfusion and increased small intestinal permeability, defined as the non-mediated diffusion of large normally restricted molecules from the intestinal lumen to the blood (Lambert 2009), have been postulated as key mechanisms (van Wijck et al. 2012). It is thought that a reduction in splanchnic blood flow may lead to damage of the intestinal epithelial cells that line the gastrointestinal tract (Zuhl et al. 2014a). Passage of ions, water and molecules through the paracellular pathway is regulated by the tight junctions of the epithelia (Gonzalez-Mariscal et al. 2003), and consequent disruption of these tight junctions during exercise can lead to increased intestinal permeability allowing passage of both small and large molecules. Increased intestinal permeability results in the translocation of endotoxins from the intestinal lumen into systemic circulation (Selkirk et al. 2008). Lipopolysaccharide (LPS) endotoxins are found in large quantities in the human gut (van Deventer et al. 1988) and increased circulating LPS levels in athletes have been found to be associated with GI symptoms including nausea, vomiting and diarrhoea (Jeukendrup et al. 2000). Increased permeability or circulating endotoxin could also impact on physical performance (Vargas and Marino 2014) or delay recovery (van Wijck et al. 2013). Recent investigations have, therefore, examined the efficacy of nutritional strategies such as colostrum, probiotics and glutamine in an attempt to lessen such gastrointestinal disruption (Marchbank et al. 2011; Shing et al. 2014; Zuhl et al. 2014b).

Glutamine is a natural non-essential amino acid and is the most abundant free amino acid in human plasma and skeletal muscle (Gleeson 2008) where it performs a number of roles including acting as fuel for cells of the gut mucosa and immune system (Fürst and Stehle 1995; Parry-Billings et al. 1992; Walsh et al. 1998). It has been proposed that permeability of the intestinal barrier increases following depletion of intestinal glutamine, whereas glutamine supplementation can restore intestinal barrier homeostasis (Camilleri et al. 2012). To this end, it is noteworthy that 7 days of glutamine supplementation at 0.9 g kg ^−1^ of fat-free mass (FFM) per day reduces intestinal permeability in humans exercising in the heat. Subsequently, it was shown that a single acute dose of glutamine (0.9 g kg ^−1^ FFM) 2 h before exercise was sufficient to attenuate the increase in intestinal permeability caused by a 60-min run in the heat (Zuhl et al. 2015), although GI symptoms were not reported. However, an acute dose of glutamine at 0.9 g kg ^−1^ of FFM still equates to 54 g for a 70-kg individual with 15% body fat, a dose that might not be practical for many athletes. To date, no study has examined if a lower dose of glutamine administered acutely can attenuate the increase in intestinal permeability observed following endurance exercise or if the effect is dose dependent. While a connection has been made between increases in endotoxaemia and GI symptoms in athletes during and following prolonged strenuous exercise (Altenhoefer et al. 2004; Brock-Utne et al. 1988; Jeukendrup et al. 2000), markers of GI permeability and symptoms are not always connected. We have recently shown that there was no connection between subjective GI symptoms and markers of GI permeability following high-intensity intermittent running (Pugh et al. 2017). This has added to the discrepancy between field studies showing increases in GI permeability and subjective symptoms, and previous laboratory studies showing increases in GI permeability but not symptoms (Lambert et al. 2008; Van Wijck et al. 2011; Zuhl et al. 2014b). Some previous studies have also not reported subjective GI symptoms at all (Marchbank et al. 2011; Zuhl et al. 2014b). Therefore, to better understand the connection between markers of gut permeability, and symptoms of GI distress, it is crucial that studies assess symptoms rather than focus purely on biological markers.

If a worthwhile attenuation of intestinal permeability could be achieved with a smaller dose of glutamine than that used previously, it could be a practical acute intervention for athletes. Therefore, the aim of the current study was to test the dose–response relationship of acute glutamine supplementation upon markers of intestinal permeability and GI symptomology in recreationally active male runners. We hypothesised that acute glutamine ingestion would reduce markers of GI permeability and damage [assessed using intestinal-fatty acid binding protein (I-FABP)], in a dose-dependent manner with the highest dose having the greatest effects. A secondary aim was to assess whether this resulted in lower subjective GI symptoms.

## Methods

### Participants

Ten recreationally active healthy males [age 24 ± 4 years, body mass 74.7 ± 8.5 kg, $\dot {V}{{\text{O}}_{{\text{2max}}}}$ 52.3 ± 5.4 mL kg^−1^ min^−1^] volunteered to participate in the study after providing informed written consent. None were taking medication (e.g. NSAIDs, antidepressants, or diuretics) or nutritional supplements and no participant reported any history of GI-related medical issues (e.g. IBS or abdominal surgery). The study was approved by the Ethics Committee of Liverpool John Moores University.

### Overview of experimental design

**Fig. 1:**
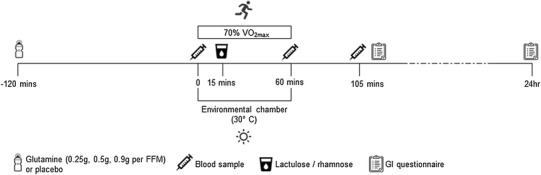
Schematic overview of the experimental protocol. Participants repeated the same protocol on four occasions separated by 1 week. In each trial participants ran at the pace set during the familiarisation session

In a 4-arm, double-blind, placebo-controlled, randomised crossover design, after baseline measures and familiarisation, each participant completed a total of four exercise trials consisting of a placebo trial and three glutamine trials at doses of 0.25, 0.5 and 0.9 g kg ^−1^ of fat-free mass (FFM). Each exercise trial consisted of a 60-min treadmill run at 70% of $\dot {V}{{\text{O}}_{{\text{2max}}}}$ in an environmental chamber set at 30 °C and a humidity range of 40–45% (relative humidity). Trials were separated by a 1-week washout period. A randomised design was used in an effort to separate the effects of glutamine supplementation from any changes that may have occurred during the testing period such as an increase in exercise capacity or acclimation to heat stress. A summary of the experimental design can be seen in Fig. 1.

### Baseline testing

During the first visit, participants were weighed and skinfold measurements were taken by an ISAK (International Society for the Advancement of Kinanthropometry) Level 1 certified anthropometrist to estimate per cent body fat and calculate FFM. Skinfold measurements were taken from four sites and body fat percentage was calculated using the Jackson and Pollock equation for men (Jackson and Pollock 1985).

Maximal oxygen consumption ($\dot {V}{{\text{O}}_{{\text{2max}}}}$) was assessed using an incremental exercise test to volitional exhaustion on a motorized treadmill (HP Cosmos, Germany). Briefly, participants started running at 10 km h^−1^, and thereafter running speed was increased by 2 km h^−1^ every 2 min. On completion of 2 min at 16 km h^−1^ the incline of the treadmill was increased by 1% every 2 min until volitional exhaustion was reached. $\dot {V}{{\text{O}}_{{\text{2max}}}}$ was validated using the following endpoint criteria: (1) RER > 1.1, (2) heart rate within 10 beats min^−1^ of age predicted maximum and (3) O_2_ consumption no longer increasing despite increased workload (Howley et al. 1995). Based on the results of the incremental test the running speed required to elicit 70% $\dot {V}{{\text{O}}_{{\text{2max}}}}$ was estimated for each participant using a linear regression equation.

Participants were asked to attend a session at the laboratory a week later to familiarise themselves with the exercise protocol. Participants began running in an environmental chamber set at 30 °C at the velocity predicted as 70% of $\dot {V}{{\text{O}}_{{\text{2max}}}}$ during baseline testing. Treadmill speed was adjusted as necessary to maintain this intensity throughout 1 h of running. In all exercise trials that followed participants ran at the pace recorded during their familiarisation session.

### Glutamine supplementation and dietary control

Prior to each trial participants were provided with an opaque bottle containing the placebo or a dose of glutamine. Glutamine was provided from a company registered with ‘Informed Sport’ to minimise the risk of supplement contamination. Participants ingested glutamine mixed with 400 mL of water and 100 mL of sugar-free lemon cordial or a placebo which was 400 mL of water and 100 mL of sugar-free lemon drink only. The drink was consumed 2 h prior to commencing exercise, and participants were informed to consume all of the drink within 5–10 min. Participants were asked to record their food intake the day before the first exercise trial and were instructed to repeat this intake the day before each subsequent visit.

### Exercise protocol

Participants reported to the laboratory at the same time of day for each trial (07:30–09:00) following an overnight fast. Baseline blood samples were taken before exercise commenced. Each exercise trial consisted of running for 60 min in an environmental chamber set at 30 °C and a humidity range of 40–45% (relative humidity). Oxygen consumption was sampled every 5 min throughout the trial using an automated gas analysis system (Oxycon Pro, Jaeger, Wuerzberg, Germany). Heart rate was recorded every 5 min with the Polar FT1 HRM (Polar Electro, Kempele, Finland). Fifteen minutes into the exercise trial, the sugar probe drink (see below) was consumed for measurement of small intestinal permeability. Participants were permitted to consume water ad libitum during and after each trial; drinking patterns were not recorded. Further venous blood samples were taken immediately post- and 45 min post-exercise. Core temperature was monitored throughout the trial using a rectal thermistor (Grant Squirrel SQ800, Cambridgeshire, UK). Trials were to be terminated early if participants reached a core temperature of 39.6 °C, but this did not occur at any time during the study. Intestinal permeability and symptomology were also measured during a resting condition, performed under the same environmental conditions as exercise trials. Timings for administration of the sugar probe and blood sampling mirrored those during the exercise trials.

### Blood analysis

Blood samples were collected into vacutainers containing EDTA, lithium heparin or serum separation tubes and stored on ice or at room temperature until centrifugation at 3000 rpm for 15 min at 4 °C. Following centrifugation, aliquots of plasma or serum were stored at − 80 °C for later analysis. Samples were analysed for plasma glutamine and I-FABP and serum IL-6. Intestinal permeability was assessed using a lactulose/rhamnose dual sugar absorption test (L:R).

### Assessment of plasma glutamine

Plasma glutamine was assessed using a quantitative colorimetric enzyme assay kit (EGLN-100, BioAssay Systems, Hayward, CA) sensitive to 0.023 mM glutamine according to manufacturer’s instructions. Samples were diluted 1:2 with distilled water. Glutamate was measured in each sample and subtracted from the glutamine absorbance of the respective sample. The coefficient of variation using this assay was 6.7%.

### Assessment of intestinal permeability

Intestinal permeability was assessed by analysing serum samples using a previously published protocol (Fleming et al. 1996), with the modification of using rhamnose instead of mannitol as the monosaccharide probe. Briefly, 15 min after exercise, a 50-mL sugar probe solution (5 g lactulose, 2 g rhamnose) was consumed and the ratio of the sugars was measured from serum samples 60 min post exercise. The respective sugars were separated using high-pressure liquid chromatography (HPLC) and quantitated by use of a pulsed electrochemical detector using a gold working electrode and silver–silver chloride reference electrode. The detection potential was − 0.01 V (0–0.5 s), the oxidation potential was + 0.75 V (0.51–0.64 s), the reduction potential was − 0.75 V (0.65–0.75 s) and the integration period was 0.05 to 0.5 s. Retention times were 2.7 min for rhamnose and 6.1 min for lactulose using 120 mmol/L NaOH as an isocratic eluant. The coefficient of variation for lactulose and rhamnose combined was 11%.

### Assessment of I-FABP

I-FABP was determined by analysis of EDTA plasma samples using an ELISA kit (Hycult Biotechnology, Uden, the Netherlands) according to the manufacturer’s instructions. I-FABP concentrations were measured in samples taken pre- and immediately post-exercise. The intra-assay coefficient of variation was 8%.

### Assessment of gastrointestinal symptoms

Global gastrointestinal symptoms were recorded every 5 min during each experimental protocol using a GI discomfort scale (adapted from Pfeiffer et al. 2009). Participants rated their symptoms on a 10-point scale, ranging from 0 (‘no problem at all’) to 9 (‘the worst it has ever been’), with a score > 4 being regarded as serious. Participants were asked to complete a more detailed questionnaire (adapted from Pfeiffer et al. 2012) post-exercise to assess any specific symptoms of GI discomfort encountered during the session, such as bloating, flatulence and urge to vomit. GI symptoms were scored on a 10-point scale (0 = no symptoms and 9 = very severe symptoms) with a score > 4 being regarded as serious. This questionnaire was completed again 24 hours later to assess GI symptoms following the exercise trial.

### Statistical analysis

Descriptive statistics (mean ± SD) were calculated for all variables. Magnitude-based inferences were then employed to determine the likelihood of meaningful changes with glutamine supplementation compared to placebo. For intestinal permeability, I-FABP and physiological measures, the mean effect and 90% confidence limits (hereafter depicted as effect; ± 90% confidence limit) were calculated. Non-clinical (mechanistic) inferences were made based on a smallest worthwhile change of 0.2 x the SD of the placebo trial with standardised changes (ES) of 0.6 and 1.2 being considered moderate and large, respectively (Hopkins 2009). Threshold probabilities for a meaningful effect based on the 90% confidence limits were as follows: <1%, almost certainly not; 1–5%, very unlikely; 5–25%, unlikely; 25–75%, possibly; 75-97.5%, likely; 97.5–99% very likely; >99%, almost certainly. Effects with > 5% confidence limits across a likely small positive or negative change were classified as unclear (Hopkins 2006). All analyses were completed using a predesigned spreadsheet (Hopkins 2006).

## Results

### Glutamine supplementation did not affect physiological or thermoregulatory responses to exercise

Participants ran at 10.1 ± 0.9 km h^−1^ at an average intensity of 72.8% ± 4.7 $\dot {V}{{\text{O}}_{{\text{2max}}}}$ across all trials. There were no clear differences in heart rate, thermal comfort, core temperature or subjective gastrointestinal comfort between doses (Table 1).

**Table 1:** Physiological responses to exercise by dose of glutamine

	Placebo	0.25 g kg^−1^	0.5 g kg^−1^	0.9 g kg^− 1^
Mean HR (%max)	82.5	83.2	84.8	83.3
	–	0.7; ± 1.6	2.3; ± 4.0	0.8; ± 2.8
Thermal comfort (AU)	7.6	7.8	7.5	7.8
	–	0.2; ± 0.5	− 0.1; ± 0.5	0.2; ± 0.4
Core temperature (˚C)	38.46	38.53	38.61	38.42
	–	0.07; ± 0.3	0.15; ± 0.3	− 0.04; ± 0.3
Gastrointestinal comfort (AU)	1.1	1.1	0.9	1.4
	–	0.0; ± 0.5	− 0.2; ± 0.7	0.5; ± 0.5

Data are mean and change in mean: ± 90% CL vs placebo

Inferences based on a smallest worthwhile change 0.20 of the between-subjects SD in placebo. Inferences were unclear vs placebo for all measures at all supplement dosages

### Glutamine supplementation elevates plasma glutamine in a dose dependent manner

**Fig. 2:**
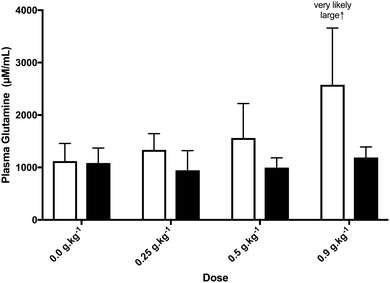
Plasma glutamine concentrations at Inference refers to meaningful change relative to Placebo trial

The effects of glutamine supplementation on plasma glutamine concentration are presented in Fig. 2. There was a very likely *large* increase pre-exercise with the 0.9 g kg^− 1^ dose compared with placebo (ES = 4.2; ± 2.7). Doses of 0.25 and 0.5 g kg^− 1^ glutamine resulted in moderate (ES = 1.1; ± 1.8) and large (ES = 1.5; ± 0.8) increases, although these were unclear. Mean plasma concentrations were lower post-exercise in all supplement trials, although all changes were unclear compared to placebo.

### Glutamine attenuates GI permeability in a dose-dependent manner

**Fig. 3:**
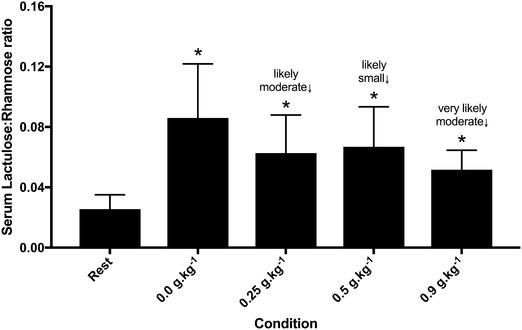
Plasma lactulose:rhamnose ratio at rest and following exercise with different acute doses of glutamine supplementation. *Large increase relative to rest. Inference refers to meaningful change relative to Placebo trial

There was a *large* increase in the L:R ratio following exercise in all trials when compared to rest. However, when compared to the placebo trial, the post-exercise L:R ratio was lower following glutamine supplementation with 0.25 g kg^−1^ (m*oderate* ES = 0.6; ± 0.5), 0.5 g kg^−1^ (small ES = 0.5; ± 0.5) and 0.9 g kg^−1^ (moderate ES = 0.9; ± 0.6) (Fig. 3).

### Larger doses of glutamine have a small modulating effect on I-FABP

**Fig. 4:**
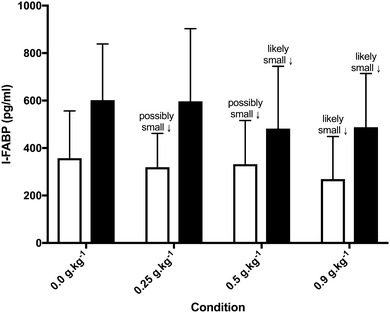
I-FABP concentrations before and pre (clear bars) and post (solid bars) exercise. Inference refers to meaningful change relative to matched time point during Placebo trial

There were possible or likely small reductions in I-FABP before exercise in supplement trials when compared to placebo (Fig. 4). Post-exercise, I-FABP was likely lower following glutamine supplementation with 0.5 g kg^− 1^ (small ES = 0.46; ± 0.54) and 0.9 g kg^− 1^ (small ES = 0.44; ± 0.42), but the change was unclear with 0.25 g kg^− 1^ (ES = 0.02; ± 0.38) compared to placebo.

### Gastrointestinal symptoms were low and unaffected by glutamine supplementation

Global GI symptoms were low in all exercising conditions (rated < 4) with no effect of glutamine supplementation at any given dose. Specific symptoms regarded as serious (i.e. rated > 4) represented 2–4% of symptoms reported after 45 mins and 3–4% after 24 h and included flatulence and the urge to defecate. All other symptoms were rated as less than 4 (Tables 2, 3).

**Table 2:** GI symptoms post-exercise, rated 0–9

	Placebo	0.25 g kg^−1^	0.5 g kg^−1^	0.9 g kg^−1^
Side stitch	0 (0–0)	0 (0–3)	0 (0–5)	0 (0–1)
Bloating	0 (0–2)	0 (0–2)	0 (0–1)	0 (0–2)
Urge to defecate	1 (0–5)	1.5 (0–5)	0 (0–3)	0.5 (0–4)
Diarrhoea	0 (0–5)	0 (0–5)	0 (0–0)	0 (0–1)
Flatulence	0.5 (0–2)	0 (0–1)	0 (0–2)	0.5 (0–6)
Stomach cramps	0 (0–0)	0 (0–2)	0 (0–1)	0 (0–2)
Stomach upsets	0 (0–1)	0 (0–1)	0 (0–3)	0 (0–2)
Intestinal cramps	0 (0–0)	0 (0–1)	0 (0–2)	0 (0–2)
Urge to burp	1 (0–5)	0.5 (0–3)	1 (0–2)	0 (0–2)
Nausea	0 (0–0)	0 (0–1)	0 (0–5)	0 (0–6)
Urge to vomit	0 (0–0)	0 (0–0)	0 (0–0)	0 (0–5)
Dizziness	0 (0–3)	0.5 (0–5)	0 (0–2)	0 (0–5)
Shivering	0 (0–0)	0 (0–2)	0 (0–0)	0 (0–0)
Heart burn	0 (0–2)	0 (0–0)	0 (0–0)	0 (0–0)

Data are median and range appearing in parenthesis

**Table 3:** GI symptoms 24-h post-exercise rated 0–9

	Placebo	0.25 g kg^−1^	0.5 g kg^−1^	0.9 g kg^−1^
Side stitch	0 (0–0)	0 (0–0)	0 (0–0)	0 (0–0)
Bloating	0 (0–3)	0 (0–2)	0 (0–1)	0 (0–1)
Urge to defecate	1 (0–3)	2 (0–5)	2 (0–3)	2 (0–5)
Diarrhoea	0.5 (0–2)	0 (0–1)	0 (0–1)	0 (0–1)
Flatulence	2.5 (0–8)	1.5 (0–4)	1 (0–5)	1 (0–6)
Stomach cramps	0 (0–2)	0 (0–2)	0 (0–2)	0 (0–0)
Stomach upsets	0 (0–5)	0 (0–4)	0 (0–0)	0 (0–0)
Intestinal cramps	0 (0–4)	0 (0–1)	0 (0–0)	0 (0–0)
Urge to burp	2 (0–5)	0 (0–3)	1 (0–5)	0 (0–4)
Nausea	0 (0–0)	0 (0–7)	0 (0–0)	0 (0–2)
Urge to vomit	0 (0–0)	0 (0–7)	0 (0–0)	0 (0–0)
Dizziness	0 (0–5)	0 (0–4)	0 (0–0)	0 (0–0)
Shivering	0 (0–0)	0 (0–0)	0 (0–0)	0 (0–0)
Heart burn	0 (0–0)	0 (0–1)	0 (0–0)	0 (0–0)

Data are median and range appearing in parenthesis

## Discussion

The aim of the present study was to assess the effects of various doses of acute glutamine supplementation on markers of GI permeability and damage prior to and following a bout of endurance exercise in the heat. While it has been previously shown that a large, acute dose of glutamine (0.9 g kg^−1^ FFM) is able to attenuate exercise-induced increases in intestinal permeability (Zuhl et al. 2015), we report for the first time that lower doses (as low as 0.25 g kg^−1^) ameliorates this effect in what appears a dose–response manner. In addition, we show that glutamine of at least 0.5 g kg^−1^ can also attenuate exercise-induced increases in I-FABP. Nonetheless, subjective symptoms of gastrointestinal symptoms immediately and 24 h after exercise in this sample were low, and glutamine had no modulatory effect. Taken together, our data suggest that acute glutamine supplementation (even at a low dose of 0.25 g kg^−1^) can reduce GI permeability and damage post exercise; however, its use to reduce mild GI symptoms that are typically associated with endurance exercise in the heat could not be elucidated in the present study. Athletes competing in the heat may, therefore, benefit from acute glutamine supplementation prior to exercise in order to maintain gastrointestinal integrity.

Numerous exercise protocols are used to model the exercise-induced increase of GI permeability compared to resting values (Marchbank et al. 2011; Pals et al. 1997; Van Wijck et al. 2011). Running for 60 min has led to increases in GI permeability 1.4–1.7 times that of resting values (Lambert et al. 2008). This is exacerbated to values of 3 times resting figures when this protocol is completed in the heat (Zuhl et al. 2015), which is in agreement to values found in the current study. Glutamine is a major substrate for proliferation and differentiation of intestinal epithelial cells (Newsholme et al. 2003) and a significant body of research exists showing that glutamine supplementation is able to attenuate increases in stress-induced GI permeability (Rao and Samak 2012). We have shown that all exercise trials resulted in *large* increases in GI permeability, relative to rest. Furthermore, relative to placebo, consumption of supplemental glutamine 2 h before exercise resulted in attenuated GI permeability in what appears to be a dose-dependent manner. The apparent dose–response effect shown was also observed in post exercise I-FABP. Previously 0.9 g kg^−1^ of glutamine administered for 7 days and as a single, acute dose 2 h before exercise has been found to attenuate increases in GI permeability by around 33% (Zuhl et al. 2014b, 2015). We show a similar (40%) attenuation with this same dose and also add novel data showing that smaller doses, as low as 0.25 g kg^−1^, can also modulate this disruption, although the magnitude of effect is reduced, with approximately 25% attenuation.

While we have shown that acute glutamine supplementation can attenuate exercise-induced GI permeability, the mechanism cannot be elucidated from the present data. Up to 63% of the variance in intestinal permeability following running exercise has been attributed to changes in core temperature (Pires et al. 2016). In the current study, there was no difference in physiological response, including peak core temperature and thermal comfort between conditions, suggesting an alternate mechanism One mechanism proposed is through the activation of heat shock protein 70 (HSP70), heat shock protein-1 (HSP-1) and heat shock protein 72 (Wischmeyer 2002; Zuhl et al. 2014b, 2015). It has also been shown, in an animal model, that glutamine administration increases HSP 72 expression in a dose-dependent manner and may explain the dose–response effect on GI permeability here.

Although GI permeability and I-FABP increased during all exercise trials, ratings of GI discomfort, either during or in the following 24 h after exercise, were low to mild (all median values < 4). The low scores for GI discomfort may be because the cohort consisted of healthy males, who were well hydrated and had no history of GI disease. This is consistent with much of the recent laboratory-based research into single exercise sessions and markers of GI permeability and damage. Many of these have reported measures of increased L:R or I-FABP, but reported either low or mild scores of GI discomfort during acute exercise bouts (Lambert et al. 2008; Van Wijck et al. 2011) or have not reported GI symptoms at all (Marchbank et al. 2011; Zuhl et al. 2014b). Even during, and following, 60 min of running in the heat, 30 min of which was a distance time trial, GI symptoms were rated as “very mild” to “noticeable” (Morrison et al. 2014). This apparent discrepancy in symptom expression between field and laboratory studies may be due to several factors. Exercise modalities used in laboratory studies have often been shorter in duration and lower in relative intensity than those typically seen in competitive endurance races. Competitive events may also cause increases in mental stress not seen in laboratory studies which could exacerbate GI symptoms due to further decreases in splanchnic blood flow (Murray 2006), direct changes to intestinal bacterial composition (Palma et al. 2014) or effects on GI transit time via the central nervous system (Brouns and Beckers 1993). There may also be specific nutritional strategies employed during competition that lead to GI symptoms, that are not used during the training cycle, such as carbohydrate loading and/or carbohydrate ingestion during exercise (de Oliveira and Burini 2011). Further investigations should investigate whether there are unique aetiologies or not relating to different exercise modalities (i.e. long duration, steady state vs shorter, high intensity) and between training and competition. Finally, it may also be that the lack of relationship between symptoms and markers of permeability are related to differences between the size of the molecules used to asses permeability (typically < 0.5 kDa) and those antigens and macromolecules which may cause symptoms (typically > 10 kDa) (Menard et al. 2010).

In summary, we have confirmed that 0.9 g kg^−1^ of acute glutamine supplementation not only attenuates GI permeability relative to placebo, but also provides novel data highlighting doses as low as 0.25 g kg^−1^ could have some benefit. In order to better inform practical application, future studies should compare different doses prior to exercise of higher intensities or longer duration, particularly as these are more often associated with subjective symptoms. It is also important that future studies continue to assess subjective symptoms of GI discomfort during, and following endurance exercise alongside markers of GI permeability and damage, to better understand potential aetiologies. Therefore, while we cannot elucidate any effect on GI symptoms, athletes competing in the heat may still benefit from acute glutamine supplementation prior to exercise in order to maintain gastrointestinal integrity.

## Abbreviations

ELISA: Enzyme-linked immunosorbent assay
GI: Gastrointestinal
HR: Heart rate
I-FABP: Intestinal-fatty acid binding protein
L:R: Lactulose:rhamnose ratio
NSAID: Non-steroidal anti-inflammatory drug
RPE: Ratings of perceived exertion
TC: Thermal comfort
